# p53 expression but not p16^INK4A^ correlates with human papillomavirus-associated esophageal squamous cell carcinoma in Kazakh population

**DOI:** 10.1186/s13027-016-0065-x

**Published:** 2016-04-13

**Authors:** Lianghai Wang, Jing Li, Jun Hou, Man Li, Xiaobin Cui, Shugang Li, Xiaodan Yu, Zhiyu Zhang, Weihua Liang, Jinfang Jiang, Lijuan Pang, Yunzhao Chen, Jin Zhao, Feng Li

**Affiliations:** Department of Pathology and Key Laboratories for Xinjiang Endemic and Ethnic Diseases, Shihezi University School of Medicine, Shihezi, Xinjiang China; Department of Immunology, Shihezi University School of Medicine, Shihezi, Xinjiang China; Department of Preventive Medicine, Shihezi University School of Medicine, Shihezi, Xinjiang China; Department of Pathology, Beijing Chaoyang Hospital, Capital Medical University, Beijing, China

**Keywords:** p16^**INK4A**^, p53, Human papillomavirus, Esophageal squamous cell carcinoma, Kazakh

## Abstract

**Background:**

p16^INK4A^ expression has been used as a surrogate marker for human papillomavirus (HPV) infection in cervical cancer and head and neck cancer. p53 has also been reported as a feasible marker to identify HPV-positive oropharyngeal carcinoma and penile lesions. This study aimed to investigate p16^INK4A^ and p53 expression levels and their correlation with HPV status and clinical parameters in Kazakh patients with esophageal squamous cell carcinoma.

**Methods:**

Immunohistochemical expression of p16^**INK4A**^ and p53 were evaluated in 163 cases of esophageal squamous cell carcinoma in Kazakh patients. The presence of HPV DNA was detected by polymerase chain reaction.

**Results:**

p16^**INK4A**^-positive expression was detected in 19.0 % of patients, and its expression was significantly correlated with a lower frequency of lymph node metastasis (*p* = 0.038). By contrast no significant association was found between p16^**INK4A**^-positive expression and HPV status (correlation coefficient = -0.062, *p* = 0.499). p16^**INK4A**^-positive expression did not affect the odds of tumors being HPV positive (odds ratio [OR] = 0.727 with 95 % confidence interval [CI] = 0.288–1.836). The sensitivity of p16^**INK4A**^-positive expression as an HPV marker was 0.164, with a specificity of 0.788 and a positive predictive value of 0.391. p53-positive expression was present in 88.3 % of all cases. Although no significant correlation with available clinical parameters was found, a significantly inverse correlation was observed between p53 expression and HPV status (correlation coefficient = -0.186, *p* = 0.039). Moreover, p53-positive expression decreased the odds of tumors being HPV positive (OR = 0.292 with 95 % CI = 0.086–0.990). The sensitivity of p53-negative expression as an HPV marker was 0.179, with a specificity of 0.940 and a positive predictive value of 0.714. The overall HPV prevalence was high (45.5 %) in Kazakh patients, with no significant association between HPV positivity and available clinical parameters or combined p16^**INK4A**^/p53 expression.

**Conclusions:**

p16^**INK4A**^-positive expression was associated with lymph node metastasis. Results indicate that p53-negative expression and not p16^**INK4A**^-positive expression may be used as a marker for HPV status in ESCC; however, this finding requires further studies for validation.

## Background

Esophageal cancer (EC), is one of the most common malignancies and the sixth most frequent cause of cancer-related death worldwide with a global number of 400,000 deaths in 2012 (http://globocan.iarc.fr/Default.aspx). China is one of the geographical regions with the highest incidence of esophageal cancer. Of the two main histological types, the squamous cell carcinoma (ESCC) and the adenocarcinoma (EAC), the former is the predominant type in China accounting for more than 80 % of cases [1]. The Kazakh population, which is a nomadic tribe and mainly residing in Xinjiang, Northwestern China, shows higher ESCC incidence and mortality compared with other ethnic groups in China [2]. A better understanding of ESCC pathogenesis for early diagnosis is crucial considering the present difficulties in detecting early symptoms, identifying unfavourable prognosis, and low 5-year survival rate.

Human papillomavirus (HPV) are small circular non-enveloped double-stranded DNA viruses exhibiting strict epithelial tropism and infecting either mucosa (α-papillomaviruses) or skin (β- and γ-papillomaviruses) [3]. Several studies have demonstrated that HPV infection is the main cause of cervical cancer and head and neck cancer [4, 5]. HPV infection in esophageal cancer was first reported in 1982 based on histological observations [6]. The HPV16 and HPV18 were the viral genotypes most frequently identified in the majority of the studies [7, 8]. Few studies searched for mucosal as well as cutaneous HPV sequences in esophageal lesions [9]. Recently it has been reported that the HPV prevalence in ESCC of Kazakh patients ranged from 30 % to 66.67 % [10–12]. For such reason the identification of HPV status in the ESCC lesions is important to establish their etiology and prognostic significance [13].

HPV E6 and E7 oncoproteins are able to inactivate p53 and pRb oncosuppressors, respectively, interfering with cell cycle control. The oncosuppressor pRB is a negative regulator of the cyclin-dependent kinase inhibitor p16^**INK4A**^ and its degradation mediated by HPV E7 causes the abnormal p16^**INK4A**^ and p53 expression. Surrogate markers of HPV infection are essential for cancer screening given the low cost and high sensitivity of immunohistochemistry (IHC). Studies have shown that p16^INK4A^ expression measured by IHC correlates with the presence of HPV DNA and thus can be used as a surrogate marker of HPV infection in squamous cell carcinoma (SCC) of the cervix, vagina, and oropharynx [14–17]. p53 has also been reported as a useful marker for identifying HPV-positive oropharyngeal carcinoma [18] and penile lesions [19]. However, the biomarkers of HPV status in ESCC should be further validated to improve their use for diagnosis and treatment.

This study aimed to investigate p16^INK4A^ and p53 expression levels and their correlation with HPV status and clinical parameters in Kazakh patients to raise the possibility of using them as surrogate markers of HPV infection in ESCC.

## Methods

### Patients and samples

All formalin-fixed paraffin-embedded tissue (FFPE) blocks from 163 Kazakh patients who underwent esophagectomy without prior chemotherapy or radiotherapy were obtained from the First Affiliated Hospital of Shihezi University School of Medicine, the People’s Hospital of Xinjiang Uyghur Autonomous Region, and Xinjiang Yili Prefecture Friendship Hospital in Northwestern China from 1984 to 2013. Detailed clinical data [17] of all the patients, including gender, age, differentiation, invasion depth, lymph node metastasis, and UICC stage (TNM stage), were also collected. The ESCC patients were staged according to the Cancer Staging Manual of the American Joint Committee on Cancer. The research protocol used in this study was in accordance with the medical ethics and human clinical trial committee of the Shihezi University School of Medicine, and all recruited subjects were enrolled with written informed consent.

### Tissue microarray construction

All tissues were sectioned and stained with hematoxylin and eosin. Furthermore, the morphologically representative tissue areas of each sample identified from these stained slides were marked. Subsequently, the fields corresponding to these selected regions were located in the paraffin block for tissue microarray (TMA) construction. 1.0 mm diameter tissue cylinders were punched from these areas of each donor tissue block and brought into a recipient paraffin block using a homemade semi-automated tissue arrayer (Alphelys, Plaisir, France). The region of each tissue cylinder was reviewed to guarantee that at least 70 % represented the typical region of interest in that sample. Finally, 5 μm-thick serial sections were prepared from the TMA blocks for immunohistochemical staining.

### Immunohistochemistry

Immunohistochemical staining of p16^**INK4A**^ and p53 were performed using an automated immunostainer (BOND-MAX, Leica). Slides were de-paraffinized using a bond dewax solution (29490, Leica) and rehydrated in absolute alcohol. Afterward, bond epitope retrieval solution 2 (ER20134, Leica) was used for epitope retrieval, and the slides were incubated for 20 min at 100 °C and 12 min at room temperature. The slides were washed with bond wash solution (W0080, Leica) for 3 min. Endogenous peroxidase activity was abolished by incubating the slides for 5 min in a peroxidase-blocking solution. A total of 150 μl of the primary antibody against p16^**INK4A**^ (ZM-0205, dilution 1:500, ZSGQ-BIO) or p53 (DO-7, dilution 1:600, Gene Tech) was dropped onto each slide, followed by incubation for 15 min. After posting primary and polymery for 8 min each, the slides were incubated in DAB buffer for 5 min and then washed with distilled water. Subsequently, hematoxylin was added onto each slide followed by incubation for 5 min. The slides were dehydrated in graded alcohol to xylene and mounted on an anti-fade mounting medium with mounting glass. p16^**INK4A**^-positive cervical cancer and p53-positive esophageal cancer tissues were used as positive controls. The data of p16^**INK4A**^ immunohistochemistry were available in 158 patients, whereas p53 expression was evaluable in 163 patients. Other samples were not evaluated because of the lack of carcinoma in the residual tissue material.

All the immunostained slides were independently evaluated by two experienced pathologists. Cases in which the two pathologists disagreed on the immunostaining results, a third pathologist was consulted to analyze the staining. The expressions of these two markers were scored based on cytoplasmic/nuclear staining intensity and percentage of positively stained cells. The staining intensity was categorized as follows: 0, negative; 1, buff; 2, yellow; and 3, brown. The percentages of positive stained cells were scored as follows: 0 (<5 % positive cells), 1 (6 %–25 % positive cells), 2 (26 %–50 % positive cells), 3 (51 %–75 % positive cells), or 4 (≥76 % positive cells). p16^**INK4A**^ was considered positive if strong and diffuse staining was present in >50 % of the tumor cells [20, 21].

The percentages of positive stained cells and the staining intensities were further multiplied to generate the immunoreactivity score for each case and evaluate p53 expression. Four categories of expression were listed as follow: − (a score of 0–1), + (a score of 2–4), ++ (a score of 5–8), and +++ (a score of 9–12) [22]. p53 was considered negative when the score was between + and ++ categories, whereas the +++ score and null expression category were considered p53 positive [18].

### DNA preparation and quality control

The FFPE samples were collected in 5 μm thick sections with 10–15 slides per sample for genomic DNA extraction using QIAamp DNA FFPE Tissue Kit (Qiagen, Hilden, Germany) according to the manufacturer’s instructions. The methods used were as previously described [23] to minimize the possibility of cross-contamination. Subsequently, 75 % medicinal alcohol was used to disinfect the blade before sectioning each sample, and paraffin-only samples were cut to act as no contamination control for every five samples. DNA extraction, polymerase chain reaction (PCR) amplification, and PCR product detection were performed in separate spaces. As an internal control, the quality of the prepared DNA was validated through PCR with a human β-globin (forward: 5′-CAGACACCATGGTGCACCTGAC-3′ and reverse: 5′-CCAATAGGCAGAGAGAGTCAGTG-3′). The DNA with sufficient quality was chosen for further study.

### HPV detection

HPV DNA was detected as previously described [12]. Non-degenerate primer sets GP5+/6+ (forward: TTGGATCCTTTGTACTGTGGTAGATACTAC and reverse: TTGGATCCGAAAAATAAACTGTAAATCATATTC) amplifying a 150 bp fragment within L1 gene of a wide range of HPV types were used. HPV16 E7 gene was amplified with forward primer GATGAAATAGATGGTCCAGC and reverse primer GCTTTGTACGCACAACCGAGC. A total of 5 μL of extracted DNA was amplified in a final volume of 25 μL for each PCR reaction. The reaction was performed on Life technology under the following conditions: at 95 °C for 10 min, followed by 40 cycles of denaturation at 94 °C for 30 s, annealing at 42 °C for 90 s, and extension at 72 °C for 30 s, with a final extension at 72 °C for 5 min. The assays of the samples were run in triplicate with positive and negative controls. Subsequently, the 10 % DNA sequence of the positive products was identified using NCBI Blast (www.ncbi.nlm.nih.gov/BLAST) to confirm the HPV type detected by PCR.

### Statistical analysis

All statistical analyses were performed using SPSS Statistics 17.0 software. Associations among p16^**INK4A**^, p53 expression, HPV status, and clinical parameters were analyzed using *χ*^2^-test or Fisher’s exact test. Spearman’s rank correlation coefficients were analyzed to investigate the possible correlations between HPV status and p16^**INK4A**^ and p53 expression levels. All statistical tests were two-sided and *p*-values considered significant when *p* < 0.05.

## Results

### Immunohistochemical expression of p16^INK4A^ in correlation with clinical parameters and HPV status in Kazakh patients with ESCC

**Fig. 1:**
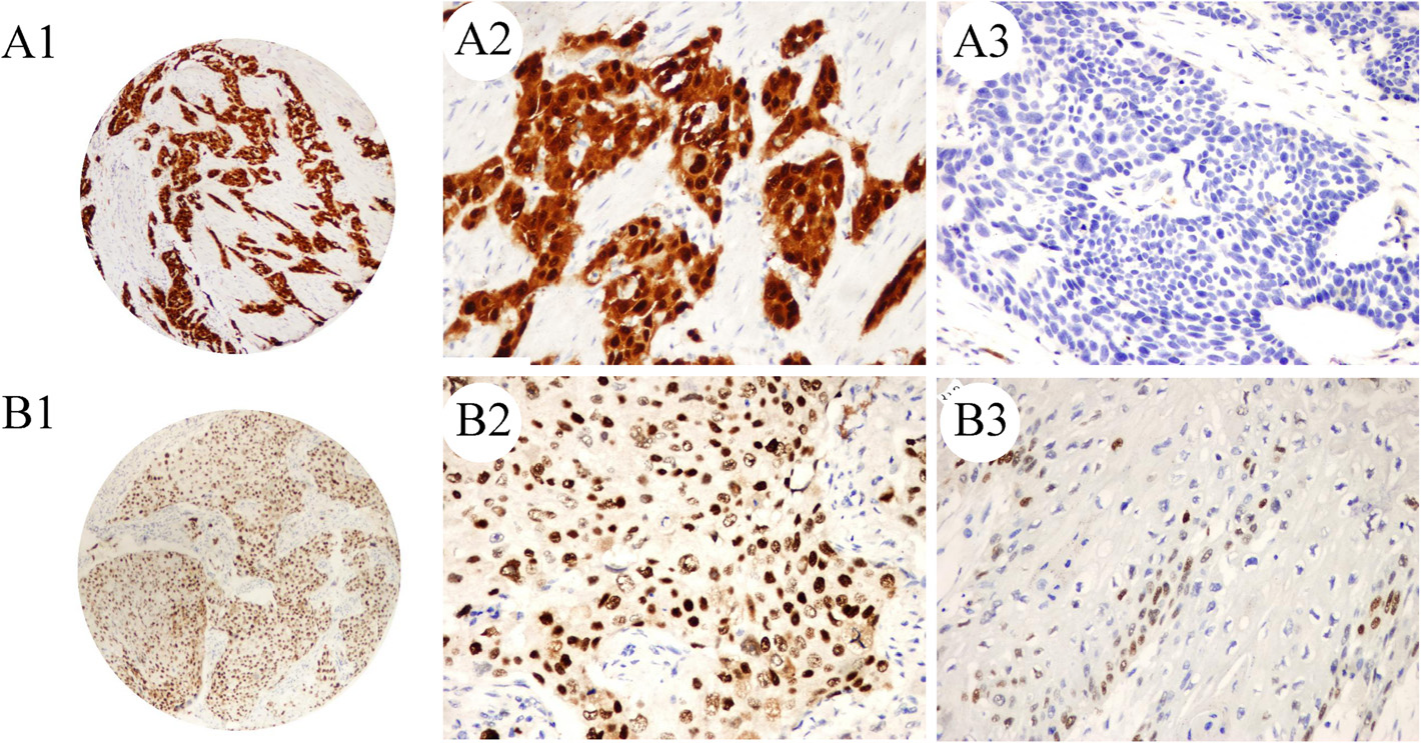
Immunohistochemical staining of p16 and p53 in Kazakh ESCC tissues. High p16 and p53 expression levels in ESCC (**A1**, p16; **B1**, p53; original magnification 40×). High power view (original magnification 200×) shows positive staining for p16 and p53 in the nucleus/cytoplasm and nucleus staining of cancer cells, respectively (**A2**, p16; **B2**, p53) and p16- and p53-negative expression (**A3**, p16; **B3**, p53; original magnification 200×)

A total of 158 ESCC samples were analyzed for p16^**INK4A**^ expression through immunohistochemistry, with 30 (19.0 %) and 128 (81.0 %) showing positive and negative expressions, respectively (Fig. 1). A significant inverse correlation was observed between p16^**INK4A**^ expression and lymph node invasion (*p* = 0.038). Patients with p16^**INK4A**^-positive expression had significantly less lymph node metastasis (35.7 % versus 57.5 %) and were diagnosed at less advanced TNM stage, although the difference was not significant (*p* = 0.147). No correlation was found between p16^**INK4A**^ expression and gender, age at diagnosis, histopathological grade, and invasion depth (Table 1).

**Table 1:** p16^**INK4A**^ expression in correlation with clinical parameters and HPV status in Kazakh patients with ESCC

Clinical properties	Total	p16 expression (n/%)		
		Negative (%)	Positive (%)	*P* value
Gender				
Male	103	85(66.4 %)	18(60.0 %)	0.507
Female	55	43(33.6 %)	12(40.0 %)	
Age (mean)				
< 58	85	60(48.0 %)	13(50.0 %)	0.853
≥ 58	67	65(52.0 %)	13(50.0 %)	
Histopathological grade				
Well	38	34(27.2 %)	4(13.3 %)	0.268
Moderate	97	76(60.8 %)	21(70.0 %)	
Poor	20	15(12.0 %)	5(16.7 %)	
Invasion depth				
T1-T2	78	63(53.8 %)	15(51.7 %)	0.838
T3-T4	68	54(46.2 %)	14(48.3 %)	
Lymphatic invasion				
N0	66	48(42.5 %)	18(64.3 %)	0.038
N1-N3	75	65(57.5 %)	10(35.7 %)	
TNM Staging				
I/II	76	59(60.8 %)	17(77.3 %)	0.147
III/IV	43	38(39.2 %)	5(22.7 %)	
HPV infection				
Negative	66	52(53.1 %)	14(60.9 %)	0.499
Positive	55	46(46.9 %)	9(39.1 %)	

*P* < 0.05 indicates a significant association among the variables

Although p16^**INK4A**^ is used as a surrogate marker for HPV-associated cervical cancer and head and neck cancer, the prevalence of HPV infection in p16^**INK4A**^-positive and p16^**INK4A**^-negative specimens demonstrated no statistically significant difference (correlation coefficient = -0.062, *p* = 0.499). p16^**INK4A**^-positive expression did not affect the odds of tumors being HPV positive (odds ratio [OR] = 0.727 with 95 % confidence interval [CI] 0.288–1.836). The sensitivity of p16^**INK4A**^-positive expression as an HPV marker was 0.164, with specificity of 0.788 and positive predictive value of 0.391.

### Immunohistochemical expression of p53 in correlation with clinical parameters and HPV status in Kazakh patients with ESCC

A total of 163 ESCC cases were included in this analysis, of which 144 (88.3 %) and 19 (11.7 %) exhibited p53 positive and negative expression, respectively (Fig. 1). Patients with p53 positive expression were diagnosed at a younger age (53.6 % versus 36.8 %) and with moderate to poor histopathological grade (77.1 % versus 57.9 %), although the difference was not significant (*p* = 0.171 and 0.070, respectively). No correlation was detected between p53 expression and other clinical parameters (Table 2).

**Table 2:** p53 expression in relation to clinical parameters and HPV status in Kazakh patients with ESCC

Clinical properties	Total	p53 expression (n/%)		
		Negative (%)	Positive (%)	*P* value
Gender				
Male	106	10(52.6 %)	96(66.7 %)	0.228
Female	57	9(47.4 %)	48(33.3 %)	
Age (mean)				
< 58	82	7(36.8 %)	75(53.6 %)	0.171
≥ 58	77	12(63.2 %)	65(46.4 %)	
Histopathological grade^a^				
Well	40	8(42.1 %)	32(22.9 %)	0.070
Moderate	98	8(42.1 %)	91(65.0 %)	
Poor	20	3(15.8 %)	17(12.1 %)	
Invasion depth				
T1-T2	76	8(47.1 %)	68(51.1 %)	0.752
T3-T4	74	9(52.9 %)	65(48.9 %)	
Lymphatic invasion				
N0	68	6(33.3 %)	62(49.2 %)	0.207
N1-N3	76	12(66.7 %)	64(50.8 %)	
TNM Staging				
I/II	79	9(60.0 %)	70(64.2 %)	0.750
III/IV	45	6(40.0 %)	39(35.8 %)	
HPV infection				
Negative	67	4(28.6 %)	63(57.8 %)	0.039
Positive	56	10(71.4 %)	46(42.2 %)	

*P* < 0.05 indicates a significant association among the variables

^a^ Well differentiation vs. moderate + poor differentiation

A significantly inverse correlation between p53 expression and HPV status was found (correlation coefficient = -0.186, *p* = 0.039). The prevalence of HPV DNA in p53-negative specimens was significantly increased compared with that in p53-positive tumors (71.4 % versus 42.2 %). p53-positive expression decreased the odds of tumors being HPV positive (OR = 0.292 with 95 % CI = 0.086–0.990). The sensitivity of p53-negative expression as an HPV marker was 0.179, with specificity of 0.940 and positive predictive value of 0.714.

### Correlation of HPV status with clinical characteristics and p16^INK4A^ and p53 expression levels

Among the 123 Kazakh patients with ESCC evaluated for HPV status 56 (45.5 %) were found HPV-positive and 67 (54.5 %) HPV-negative (Table 3). No significant differences were observed between HPV positivity and gender, patient age, tumor differentiation, invasion depth, lymph node metastasis, or TNM stage.

**Table 3:** Correlation between HPV status and clinical parameters in Kazakh patients with ESCC

Clinical properties	Total	HPV status (n/%)		
		Negative (%)	Positive (%)	*P* value
Gender				
Male	78	42(62.7 %)	36(64.3 %)	0.855
Female	45	25(37.3 %)	20(35.7 %)	
Age (mean)				
<57	55	29(43.9 %)	26(47.3 %)	0.714
≥57	66	37(56.1 %)	29(52.7 %)	
Histopathological grade^a^				
Well	28	15(22.4 %)	13(23.6 %)	0.937
Moderate	77	42(62.7 %)	35(63.6 %)	
Poor	17	10(14.9)	7(12.7 %)	
Invasion depth				
T1-T2	59	37(55.2 %)	22(40.0 %)	0.094
T3-T4	63	30(44.8 %)	33(60.0 %)	
Lymphatic invasion				
N0	55	28(43.8 %)	27(50.9 %)	0.438
N1-N3	62	36(56.2 %)	26(49.1 %)	
TNM Staging				
I/II	74	40(61.5 %)	34(65.4 %)	0.668
III/IV	43	25(38.5 %)	18(34.6 %)	

^a^ Well differentiation vs. moderate + poor differentiation

The relationship between HPV status and p16^**INK4A**^ or p53 expression levels has been analyzed in order to identify possible associations. Previous studies reported that high-risk HPV16 was the predominant genotype in patients with ESCC among the Kazakh populations [24, 25], therefore we stratified the results in HPV16 positive cases to see if there was any association with the expression of p16^**INK4A**^ and p53. Results showed that HPV16 infection was associated with p53 (*p* = 0.012) but not with p16^**INK4A**^ expression (*p* = 0.987). Similar results were obtained considering all HPV infections. p16^**INK4A**^ and p53 expression patterns exhibited a slight inverse correlation, but this correlation was not statistically significant (correlation coefficient = -0.061, *p* = 0.455, Table 4). Furthermore, the combined p16^**INK4A**^/p53 expression was not significantly correlated with HPV status in Kazakh patients with ESCC (correlation coefficient = -0.077, *p* = 0.420, Table 5).

**Table 4:** Correlation between p16^**INK4A**^ and p53 expression in Kazakh patients with ESCC

	p16^-^	p16^+^	Correlation coefficient	*P* value
p53^-^	14	5	-0.061	0.455
p53^+^	107	25

**Table 5:** Correlation between p16^**INK4A**^/p53 expression and HPV status in Kazakh patients with ESCC

	HPV status		Correlation coefficient	*P* value
	Negative	Positive		
p16^+^ p53^+^	12	7	-0.077	0.420
p16^-^ p53^-^	2	8		
p16^+^ p53^-^	2	2		
p16^-^ p53^+^	48	32		

## Discussion

We have evaluated the HPV status and p16^**INK4A**^ and p53 expression levels in ESCC from Kazakh patients. One limitation of our study is the relative small sample size. Nevertheless, this is among the largest studies addressing p16^**INK4A**^ and/or p53 expression and HPV infection in ESCC of Kazakh population [26, 27].

The use of p16^**INK4A**^ immunohistochemical analysis as a surrogate marker of HPV infection in squamous cell carcinoma of the cervix, vagina, and oropharynx has been supported by many studies in recent years [15, 17, 28–30]. The p16^**INK4A**^ expression is indicative of high risk HPV infection in cancers of squamous cell origin [31]. In our study, patients with p16^**INK4A**^ overexpression have a better prognosis, are correlated with less lymph node metastasis (*p* = 0.038), and are frequently associated with lower-grade TNM stage (*p* = 0.147), which are in accordance with previous studies [21, 26, 32]. Furthermore, p16^**INK4A**^ positivity has been detected in 16.4 % of HPV-positive patients with ESCC, which is lower than previously published data reporting a range of prevalence between 20 % and 86.2 % [26, 33, 34]. In addition, a correlation between p16^**INK4A**^ overexpression and HPV DNA positivity was previously found in HPV-related oropharynx carcinoma [28, 29, 35]. This association has also been previously reported in ESCC [26, 36]. However, in the present study the p16^**INK4A**^ over expression is not associated with HPV status (*p* = 0.499, OR = 0.727 with 95 % CI = 0.288–1.836). This finding is consistent which data described in a meta-analysis [37] and recent study [34]. The inconsistency may be explained by the limited number of patients included in these studies and the lack of uniformity in cut-off values (different criteria ranged from >0 % to >70 % of tumor cells displaying moderate to strong staining) to define p16^INK4A^ overexpression. In the present study, a cut-off value of 50 %, which has been validated to correlate with the presence of HPV in oropharyngeal SCC [16, 21], was utilized to evaluate p16^INK4A^ staining. The discrepant results may also be attributed to the variation in HPV prevalence because of different geographic areas and ethnicity of patients [38, 39]. In addition to previously described factors, which may influence the accuracy of p16^**INK4A**^ staining for HPV status, an aberrant p16^**INK4A**^ expression such as p16^**INK4A**^ (+)/HPV(–) and p16^**INK4A**^ (–)/HPV(+) cases in various cancers exists [15, 17, 40, 41]. Many of tumors with high p16^INK4A^ expression were HPV-negative indicating that non-HPV factors also lead to p16 overexpression in ESCC. The diametrical expression of p16^**INK4A**^ may be caused by different genetic alterations. For example, 11q is frequently detected to be gained in HPV-negative oropharyngeal SCC, wherein Ets (a protein that can raise the p16^**INK4A**^ level) is located [15, 42]. Rb1 alterations and subsequent p16 ^**INK4A**^ overexpression have also been described in non-HPV-driven tumors [43]. Therefore, the p16^**INK4A**^ expression in HPV-negative tumors needs to be further investigated to obtain additional information in ESCC etiology, especially in low-incidence HPV geographic regions.

Acting as a transcription factor in cell cycle regulation, genomic stability and apoptosis, p53 protein displays the highest correlation with a number of cancers [44, 45]. p53 expression may be regarded as an indicator of p53 gene mutation. p53 levels are generally low or even undetectable under normal conditions [46]. However, p53 shows nuclear staining because of the accumulation of mutant p53, which is resistant to degradation. Although accumulation of p53 identified by IHC does not necessarily indicate gene mutation, p53 overexpression in most cases (85 %) implies an underlying mutation [47]. p53 has also been reported as a feasible marker for identifying HPV-positive oropharyngeal carcinoma and penile lesions [18, 19]. In the present study, patients with p53-positive expression were younger (*p* = 0.171) and had poorer differentiation levels (*p* = 0.070) than those with p53-negative expression, although these differences were not significant. Similar to previously reported data, [27, 48], these results indicate that p53 may serve as an unfavourable prognostic marker in ESCC. Moreover, p53 expression exhibited a significantly inverse correlation with HPV status (*p* = 0.039, OR = 0.292 with 95 % CI = 0.086–0.990), which is in accordance with a previous study [49]. HPV-associated oropharyngeal SCCs generally show a low level of p53 protein because of degradation through viral E6 protein [18], whereas HPV-negative tumors show absent or high p53 protein level because of nonsense or missense p53 mutations [50]. Thus, p53 IHC may be used as a rapid, easy, and inexpensive screening test with high specificity (0.940) and high positive predictive value (0.714) for HPV in ESCC. However, p53 IHC should be evaluated in larger studies given the small number of p53-negative patients.

HPV positivity was detected in 45.5 % of ESCC patients in this study, which is similar with previously reported data in Kazakhs [10–12], with a prevalence ranging from 30 % to 66.67 %. No correlation was observed between HPV positivity and clinicopathological characteristics, which is in agreement with previous studies on ESCC [51]. However this finding is not in agreement with results obtained in cervical and head and neck cancer patients [17, 52]. Nevertheless, several studies have suggested that HPV mRNA detection may differentiate active HPV infections from inactive viruses and transient HPV contamination [53, 54]. In addition, the correlation between HPV infection and p16^**INK4A**^ and p53 co-expression did not statistically differ. Likewise, p16^**INK4A**^ and p53 expression levels have been analyzed in primary adenocarcinoma of the urinary bladder on 36 samples. A slight inverse correlation between p16^**INK4A**^ and p53 expression was observed but without statistical significance [31], which is consistent with the present results.

## Conclusions

In summary, p16^**INK4A**^-positive expression should not be interpreted as a reliable surrogate marker for HPV infection in Kazakhs with ESCC but can indicate lower risk of lymph node metastasis. Our findings show that p53 expression may be a useful biomarker for diagnosis and prognosis of HPV-positive ESCC. Nonetheless, further studies should be performed to investigate toinvestigate the molecular alterations of these cell-cycle related proteins to elucidate ESCC pathogenesis.
